# e–Mental Health Program to Prevent Psychological Distress Among French-Speaking International Students in a Linguistic-Cultural Minority Context (Ottawa, Alberta, and Quebec): Protocol for the Implementation and Evaluation of Psy-Web

**DOI:** 10.2196/47059

**Published:** 2023-09-19

**Authors:** Idrissa Beogo, Jean Ramdé, Abdoulaye Anne, Marie-Pierre Gagnon, Drissa Sia, Eric Nguemeleu Tchouaket

**Affiliations:** 1 Faculty of Health Sciences School of Nursing University of Ottawa Ottawa, ON Canada; 2 Institut du Savoir Montfort Ottawa, ON Canada; 3 Département des fondements et pratiques en éducation Université Laval Québec, QC Canada; 4 Faculté des sciences infirmières Université Laval Québec, QC Canada; 5 Département des sciences infirmières Université du Québec en Outaouais Saint-Jérôme, QC Canada; 6 Département de médecine sociale et préventive, École de santé publique Université de Montréal Montréal, QC Canada; 7 Département de gestion, d'évaluation et de politique de santé Université de Montréal Montréal, QC Canada

**Keywords:** international students, psychological distress, Covid-19 pandemic, web-based, internet-based, peer support, distress, loneliness, isolation, isolated, psychological, student, students, immigrant, immigrants, foreign, social interaction, French, design, develop, development

## Abstract

**Background:**

Based on experiences with the COVID-19 pandemic, postsecondary institutions were most affected by the restrictions. Students, especially international students, have borne the brunt associated with in-person learning restrictions imposed by public health recommendations. Canada is among the top 3 countries hosting international students (ISs), including Francophone students in provinces such as Quebec and other anglophone regions. Academic restrictions were accompanied by other measures such as quarantine, self-isolation, social distancing, and travel ban, to cite some. This has had a wide-ranging impact on these ISs. The resulting psychological distress and burden may have a much greater impact on Francophone ISs in anglophone settings, many of whom had ordinarily limited access to active offers of care in French in addition to cultural barriers and low literacy of the health care system. In order to take advantage of the effectiveness of eHealth as a pertinent and promising avenue, our project intends to build a web-based application that is cost-effective, user-friendly, anonymous, and capable to prompt interactive interventions as a first-line resource for psychological distress. In fact, internet applications have been increasingly used for the management of psychological distresses, and internet-based cognitive behavioral therapy is one of the preferred methods to prevent or control them.

**Objective:**

The aims of this study are to (1) design, implement, and maintain Psy-Web for the psychological support of ISs and (2) analyze the results of the implementation of the Psy-Web platform, the additional resources solicited, and the results obtained.

**Methods:**

This interventional project will use a sequential mixed design in the exploratory phase (phase 1) including the construction of the Psy-Web platform. A quantitative prospective component (phase 2) will include the intervention content of the Psy-Web platform. In total, 105 ISs participants (study group) and 52 ISs (control group), based on a ratio of 1:2, will be considered. The control group participants include those who did not use the web platform.

**Results:**

The project is at the data collection stage (phase 1). Psy-Web will be built in accordance with the DMAIC (Define, Measure, Analyze, Improve and Control) model with the perspective of boosting its robustness. As a first-line resource to prevent psychological distress and ultimately improve their academic performance, Psy-Web is an innovative opportunity for high education managers. The project involves a multisectoral and a multidisciplinary partnership.

**Conclusions:**

The project will develop a promising web-based solution to prevent psychological distress. Ultimately, Psy-Web will be operable in multiple languages including French.

**International Registered Report Identifier (IRRID):**

PRR1-10.2196/47059

## Introduction

### Overview

The COVID-19 pandemic has affected the operations of Francophone postsecondary institutions, the latter being at the forefront of the vitality and resilience of the French community in a linguistic minority context. As a constitutionally bilingual country, the education system should normally follow that requirement. Francophone students, in English Canada, may study in their first language at postsecondary institutions and vice versa. Indeed, Francophone postsecondary institutions outside Quebec promote the French language and culture in Canada, thanks to the Canadian Charter of Rights and Freedoms [1]. This subsequently contributes to the upward trend in international student (IS) enrolment in Canadian universities over the last decade (from 5.2% in 2009-2010 to 13.2% in 2017-2018 [2]), placing Canada among the preferred host destinations. Today, member countries of the Organisation for Economic Co-operation and Development (OECD) including the United States, the United Kingdom, and Canada remain the top host of ISs [3].

Even so, these students experience many vulnerabilities related to their status as ISs, especially given the minority context in which they live. This situation has been amplified with the sudden onset of the COVID-19 pandemic, as the latter represents the worst human health threat of modern times [4,5]. In response to this global health emergency [6,7], Canada along with the rest of the world instituted public health measures, which included widespread confinement [8]. This was accompanied by other measures such as quarantine, self-isolation, social distancing, travel ban, to name a few. These measures have had a wide-ranging impact on these ISs. Added to the health burden brought about by COVID-19 were instances of racism and xenophobia [9,10].

Brook et al's [11] review of COVID-19 notes a negative impact of midlife, self-isolation, and social distancing on psychological well-being. This impact may be more significant for ISs who are already subject to “triple-acculturative stress,” as this entails the new culture dominating the study environment, the new university, and new university supervisors [12], and adjustment challenges that combine psychosomatic symptoms [13,14] and poor academic performance [15,16]. In Canada, the burden of psychological distress may be much greater for Francophone ISs in minority settings (Francophones living in English-speaking areas). Although services in French or English are constitutionalized, the literature reports limited access to active offers of care in French. Even so, Francophone patients may experience delays in treatment, limited understanding of prescriptions, or receive misdiagnoses, because of linguistic and cultural barriers. It is also well noted that students from underrepresented backgrounds had significantly higher odds of experiencing financial hardships [17]. Despite this systemic disadvantage, ISs make little use of the support resources available to them both on campus and in the public system [18]. Smith and Khawaja [19] who analyzed acculturative stress in their review of the scientific literature on ISs, found several determinants and harmful consequences of acculturative stress. The reasons fall under cultural conflicts, communication challenges, and low literacy of the care system.

In light of the effectiveness of e–mental health platforms [20-22], prompt and interactive interventions via the internet are becoming relevant tools and first-line recourses for mitigating the psychological distress of quarantined Francophone ISs, in a linguistic-cultural minority context.

For more than a decade, the internet has been increasingly used for the management of mental health issues [21], while internet-based cognitive behavioral therapy has been favored [20,22]. This alternative is cost-effective, timely, user-friendly, anonymous, and efficient for both the prevention [23-26] and the cure [27] of mental health problems. A recent systematic review with meta-analysis reported the equivalent effects of face-to-face and web-based therapy (Hedges *g*=0.05; 95% CI 0.09-0.20) [28]. Other studies using the same methods assessed the effectiveness of CBT in anxiety therapy [22,29-31], social phobia and fear [30,32], depression [22,29,30], stress [29-31], eating disorders, or sleep disorders [31].

Considering the current status of COVID-19 and the vulnerability of ISs, this research project aims to design and implement Psy-Web, a first-line intervention platform to improve access to psychological support resources. The scope of the project is 4-fold.

### Growing Number of ISs in Canada

Estimated in 2011 at 11% of the university student population [33], ISs have grown dramatically (987.7%) between 2004 and 2019 [33,34] placing Canada in third place worldwide. For example, Université Laval has nearly 7000 ISs enrolled annually, representing 14% of the total student population. The University of Saint-Boniface, the only Francophone university in Manitoba, has 18.1%; some programs are almost entirely made up of ISs.

### Double Minority Situation

Being abroad and Francophone in Manitoba, Alberta, or Ontario, students are in a double minority context, which limits their use of services. In fact, access to active offer of care for Francophones in a linguistic minority context is limited [35,36]. This limitation is even more problematic for Francophone ISs in terms of access to health services, putting them in a double minority situation.

### Low Usage of Support Resources by ISs

Smith and Khawaja's [37] shows that ISs make little use of support resources. Reasons for this include cultural conflicts in seeking help [19,38-41], communication challenges [18], confidentiality, low literacy in the care system [42], or to avoid further stigmatization than that which they would already experience by being an IS [43]. Those who seek help usually do so for situations that are deemed urgent [39,40].

### Social Capital

The physical closure of educational institutions due to COVID-19, the suddenness of the containment measures, and the lack of a predicted exit horizon disrupts the lives of the ISs and would exacerbate the negative emotions felt [44,45]. Quarantine measures lead to financial insecurity resulting from the inability to work and receive financial support from parents, who are also confined elsewhere. Also, because of their status, they are ineligible for Canadian financial support measures (eg, Canada Emergency Benefit). This precariousness could lead to promiscuity due to the inability of some to pay their rent. For instance, to our knowledge, there are no studies that indicate an avenue for psychological interventions related to the effects of the COVID-19 quarantine on Francophone ISs in a linguistic minority context. Given the scope of the issue and the evidence in the literature, this project provides an avenue for improving the psychological well-being of Francophone ISs in Manitoba in a dual linguistic-cultural minority context.

This project will implement a free interactive web-based tool “Psy-Web,” to be developed through an agile and iterative process with ISs, and educational psychologists. Three outcomes are expected: (1) prevalence of psychological disorders, (2) intervention effects and perceived quality, and (3) acceptability and usability of Psy-Web.

The specific objectives are to (1) identify evidence-based interventions adapted to ISs experiencing stress, depression, and anxiety disorders; (2) design, implement, and maintain Psy-Web for the psychological support of ISs and; (3) analyze the results of the implementation of Psy-Web, the additional resources solicited, and the results obtained.

## Methods

### Overview

The design, implementation, and evaluation of the project are based on three strengths: (1) a multidisciplinary team that specializes in psychology, nursing, informatics, education, and immigration; (2) a partnership with postsecondary institutions and organizations in the Francophone IS community; and (3) the hosting and adaptive maintenance of Psy-Web and operational support.

### Design

This interventional project will use an exploratory sequential mixed methods approach (phase 1). Phase 2 will be run as a quantitative prospective component divided into subphases. In subphase 1, the Psy-Web platform will be built using the DMAIC (Define, Measure, Analyze, Improve and Control) model [46]. This includes pretesting the entire system (website and application) for validation. Concomitantly in subphase 2, besides data gathering, we will conduct (1) an information campaign of the project through posters in university residence halls; individual texts and emails, student union emails, and home page of partner universities’ websites; (2) a pretest of Psy-Web; and (3) prospective data compilation, biweekly evaluations, and individualized follow-ups as needed. The qualitative component will occur in the fall semester of 2023.

### Participants and Variables

Due to the interventional nature of the study, we aim for a 90% power sample to detect the true proportion of psychological distress with a 95% CI, (conservative) prevalence (π) of 50%, precision (δ) of 0.05, and confidence level (β) of 1.96: 
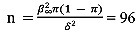
 participants. A 10% gross-up will be considered in order to substitute participants with major missing data (n=105). A control group will include 52 ISs (ratio of 1:2) who did not use the web platform or already returned to their families. We will exclude those ISs who hold a permanent resident status prior to the crisis. The reason being that permanent residents were entitled to the financial support offered to postsecondary students, and to recent postsecondary and high school graduates who were unable to find work due to the COVID-19 pandemic; the Canada Emergency Student Benefit (CESB) [47] being a prime example.

Besides the conception of the Psy-Web platform, we are ultimately targeting, through data collection, the type of psychological distress (eg, anxiety and depression) experienced as the dependent variable of interest. Independent variables will include age, gender, postsecondary program, level of education, region of origin, languages spoken, history of psychological disorders, and the coping system used. Social capital variables include institutional support programs (eg, university) and type of solidarity deployed. Immigration history and 2019 income will be the control variables.

### Psy-Web Design and Content

From needs analysis to usability assurance, the design process will incorporate consultation with 15 ISs from the University of Ottawa and Université Laval. There will be 2 stages in the design process.

First, a heuristic test known as the “Monkman heuristics” [48] and the use of the health literacy checklist [49] on the internet by 4 experts including 2 coinvestigators (MPG and IB) at each stage of the DMAIC to theoretically calibrate the website. The DMAIC cycle will optimize the efficiency, usability, and functionality of the process. The following indicators will be measured: optimization of results, aesthetics, usability, functional reliability, and sustainability [50].

Second, testing of the website with the abovementioned students. Simple classification algorithms that suggest content to the selection of symptoms listed will be preferred. At the first visit, the participant will fill in demographics, physical, mental, and social functioning, activities of daily living, and instrumental activities of daily living. Using the algorithms, selected signs and symptoms will lead to a psychoeducational activity.

### Data Collection

To ensure the representativeness of participants, maximum variation purposive sampling [51] will be used to inform the design stage (phase 1). This will consist in surveying a sample of 15 ISs from the University of Ottawa and the Université Laval. The qualitative component will be done through individual interviews (by Zoom, Zoom Video Communications) [52]. Phase 2 consists in the intervention component of the built Psy-Web platform. In total, 105 IS participants (study group) and 52 ISs (control group), based on a ratio of 1:2, will be considered. The control group participants include those who did not use the web platform. During that (testing) phase, daily, at 11 AM, the usage data of Psy-Web (ie, visits, content viewed, and ratings) and the patterns of occurrence (ie, reason and time) will be collected and discussed twice a week by the research team.

### Analysis Plan

Three phases are defined. Phase 1: analysis of automatically compiled data to assess the impact of identified issues. A biweekly assessment will be conducted by the researchers to inform interventions with the ISs. Concurrently, a historical analysis will compare pre-, peri-, and postpandemic prevalence. The quantitative analysis will include an ANOVA (or *t* test, as appropriate). Due to the categorical nature of the dependent variable, logistic regression will be preferred. A sensitivity analysis will be performed by the Generalized Estimating Equation (GEE) to test the correlated nature of the data to the region of origin [53]. A total of 94.7% of ISs are from 3 regions of Africa (West, Central, and North) with possible cultural differences. The SPSS Statistics (version 27; IBM Corp) software will be used with *P*<.05 considered significant. Phase 2 will analyze the verbatims of individual interviews using NVivo 13 (2020, R1) software. IB, JR, and MPG will thematically analyze the content according to Graneheim and Lundman [54] to capture the experience of the participants [55] and the emerging themes identified by consensus [56]. Phase 3 will conduct a sequential triangulation [57]. The acceptability and usability of Psy-Web will be assessed by the technology assessment model and system usability scale [58]. The NASSS (nonadoption, discontinuation, scaling up, diffusion and sustainability) framework of Greenhalgh et al [59] will be developed to analyze the different outcomes of the project.

### Ethics Approval

The ethics certificate has been obtained from the Research Ethics Board of Laval University (CERUL 1820). The enrollment of ISs to pilot test the Psy-Web platform has started. The recruitment is done through project website and through mouth-to-ear. An advertisement has also been posted on the faculty billboard. Recruitment enrollments are totally anonymous. No compensation is offered because we want the entire process to be anonymous, as we are dealing with a very sensitive issue. Because the questionnaires are built on Lime survey, the consent and information forms are embedded. The participant is given the choice to tick “YES,” hence consenting before moving on to completing the questionnaires. This approach of data collection and data analysis plan were discussed in June 2023 between the research team, expert ISs, and the Psy-Web platform developer for the purpose of adjustment. In early winter of 2023, we plan to disseminate the results generated nationally and internationally and to submit a manuscript to a peer-reviewed journal.

## Results

This project is at the data collection stage (phase 1). However, the website is built [60] and the core application conception is undergoing. The executive team has met in May 2023 for a follow-up. It was noticed that the initial data repository embedded on the captured Psy-Web data on a format that were difficult to retrieve. Therefore, the Université de Laval Information and Technology office suggested to bridge data repository using Institutional Lime survey package. Study recruitment started in April 2023, and data collection is expected to be concluded in January 2024. No results are available yet as it pertains to manuscript preparation.

## Discussion

This ongoing project intended ultimately to co-design and implement a first-line web-based toll, Psy-Web for the psychological support of ISs. This interactive web-based intervention project is tailored to millennials mainly and is highly relevant to addressing the psychological distress for which ISs are at high risk. The project anticipates several early benefits: (1) Using the DMAIC model, the project will coconstruct with ISs the Psy-Web application for which robustness will be data-driven. The study will capture the extent of the impact of COVID-19 quarantine among ISs at Université Laval, University of Alberta, and the University of Ottawa. Also, the platform itself offers a free, immediate, and interactive opportunity alternative (group discussion and with a psychologist) to support students. The daily collection of data and their biweekly multidisciplinary analysis constitute a continuous transfer of knowledge to inform practices and policies of dedicated universities services for ISs through Psy-Web. (2) The relevance of the project derives from the year-to-year growth of the Francophone IS population in Canada and contributes to the improvement of their quality of life and study conditions. (3) Finally, Psy-Web is a first-line resource designed to monitor and prevent psychological distress, and to ultimately improve their academic performance.

In terms of sustainability, this project will serve as a pilot for the expansion to other Francophone ISs in a Canadian minority context and beyond. The results will inform the formulation of policies related to IS issues and subsequently promote the continued attractiveness of Francophone ISs in linguistic minority settings.

## Abbreviations

CESB: Canada Emergency Student Benefit
DMAIC: Define, Measure, Analyze, Improve and Control
GEE: Generalized Estimating Equation
IS: international student
NASSS: nonadoption, discontinuation, scaling up, diffusion and sustainability
OECD: Organisation for Economic Co-operation and Development

## Data Availability

Data generated and analyzed during this study, which would be necessary to interpret, replicate, and build on the results reported in the study, will be made available to the public upon request from the Secrétariat du Québec aux relations canadiennes, the funding institution. However, this must be requested from the corresponding author as they must be stored on a secure server at Laval Université.
